# An Origami Paper-Based Analytical Device for Rapid and Sensitive Analysis of Acrylamide in Foods

**DOI:** 10.3390/mi13010013

**Published:** 2021-12-23

**Authors:** Yu Yan, Dan Zhao, Weiming Li, Xiaoqian Li, Yangyang Chang, Qiang Zhang, Meng Liu

**Affiliations:** 1 Key Laboratory of Industrial Ecology and Environmental Engineering (Ministry of Education), School of Environmental Science and Technology, Dalian University of Technology, Dalian 116024, China; 18234176390@163.com (Y.Y.); xiaoxiaodanluzd@163.com (D.Z.); liwm1990@outlook.com (W.L.); XQLi@mail.dlut.edu.cn (X.L.); yychang@dlut.edu.cn (Y.C.); 2 School of Bioengineering, Dalian University of Technology, Dalian 116024, China; zhangqiang@dlut.edu.cn

**Keywords:** acrylamide, colorimetric, enzyme linked immunosorbent assay (ELISA), paper-based analytical device

## Abstract

Rapid and sensitive detection of acrylamide in food samples is important for food safety and public health. Here, we describe a disposable origami paper-based analytical device (denoted doPAD) for colorimetric detection of acrylamide. This device uniquely exploits 3D origami folding paper for spatial control of the target recognition and signal readout, thus resulting in a positive correlation between the signals and the analytes. Under optimal conditions, the device achieved the quantitative analysis of acrylamide with a limit of detection of 1.13 μg/L within 120 min (including a derivatization time of 90 min and an assay time of 21 min). Furthermore, our method allowed the rapid and sensitive detection of acrylamide in complex food matrices. We envision that the platform described will find useful applications in the fields of food safety and environmental health.

## 1. Introduction

Acrylamide (AA) can be produced by the Maillard reaction of an amino acid, asparagine and reducing sugars (e.g., glucose, fructose) [1,2]. Under different thermal processing conditions, the AA content can vary greatly within the same food [3]. For example, high-carbohydrate foods (e.g., potatoes, cereals) processed at temperature over 120 °C contain extremely high levels of AA [3]. A large number of animal experiments showed that AA possesses reproductive toxicity, genotoxicity, and severe neurotoxicity [4,5,6]. This compound has attracted considerable attention in the fields of food safety control due to its negative consequences on health. The International Agency for Research on Cancer (IARC) has classified AA as a suspected chemical carcinogen for humans [7]. In order to limit the content of AA in food, the Commission Regulation (EU) has set the benchmark level of AA in food as 40–4000 µg kg^−1^ [8]. Hence, there is a significant need for the development of fast, sensitive, inexpensive assays for the detection of AA [9,10,11,12].

The commonly used assays for AA include gas chromatography-mass spectrometry (GC-MS) [13,14,15], and liquid chromatography-mass spectrometry (LC-MS) [16,17,18,19]. However, these methods strongly depend on instruments and have inherent disadvantages, including long detection time, complex sample pretreatment, and high cost. Furthermore, the enzyme linked immunosorbent assay (ELISA) has also been reported as a robust and inexpensive method for the detection of AA in foods [20,21]. Currently, there are some commercial ELISA kits for detecting AA based on the direct competitive ELISA. In these tests, enzyme-labeled antigens will compete with free antigens in a sample for binding to a limited amount of capture antibody attached to the surface of the slide, which would produce a target concentration-dependent signal. However, these methods usually need multiple steps and large sample volumes, hence increasing the risk of cross-contamination. In addition, there was a negative correlation between the signals and the analytes, thus potentially causing misleading results due to the high background signal [3,22,23,24,25]. Our aim of this study is to develop a powerful signal-on immunoassay toward small-molecular AA to change the signal-off situation of the traditional competitive-type immunoassays.

Paper-based analytical devices (PADs) have many advantages, being inexpensive, lightweight, biodegradable, and biocompatible [26,27]. In general, this device is fabricated by cellulose paper, which can drive the liquid without the need of external power supplies because of its natural hydrophilicity and porosity. Various patterning techniques (e.g., deposition, photolithography, and wax printing) could be used to fabricate well-defined channels on paper [28,29,30,31]. To date, PADs have evolved from two- to three-dimensional (3D) devices for wide applications in clinical diagnosis, environmental monitoring and food safety [32,33,34,35,36,37,38]. In these 3D devices, the origami design was commonly used to provide more user-friendly and multiplex testing. This setup enables the buffer to flow through the devices via capillary action, carrying different bioreagents across the folded layers and thus simplifying multi-step reactions into a single device.

Inspired by these advantages, we developed a disposable origami-based PAD (denoted doPAD) for rapid and accurate analysis of AA in food samples. We employed the concept of 3D origami folding paper to simply separate the antigen-antibody recognition and signal readout, avoiding the potential incompatibilities of reagents located in the same zone. In such way, this system produces a signal-on signal response with the increment of AA concentration in the sample. Furthermore, doPAD offers several advantages in terms of small sample volumes, simplicity and cost-effectiveness. To the best of our knowledge, such a system for quantitative determination of small molecules has not been reported so far.

## 2. Materials and Methods

### 2.1. Chemicals and Instruments

PBS buffer, tetramethylbenzidine (TMB), hydrogen peroxide (H_2_O_2_), pullulan, Tween-20 and bovine serum albumin (BSA) were obtained from Shanghai Sangon Biological Engineering Technology and Services Co., Ltd. (Shanghai, China). Nitrocellulose paper (HF120 and NC0.45 μM) were purchased from GE Healthcare (Chicago, IL, USA). The capture antibody of dAA (cAb, with a concentration of 6 mg/mL) and horseradish peroxidase-labeled acrylamide derivative (HRP-dAA, with a concentration of 2 mg/mL) were supplied by Wuxi Determine Biotechnology Co. Ltd. (Wuxi, China). All other chemicals were obtained from Sigma-Aldrich and used without further purification. Hydrophobic barrier on paper was printed using a Xerox ColorQube 8570N solid wax ink printer (Xerox Corporation, Norwalk, CT, USA).

### 2.2. Buffers Used in This Work

1 × PBS buffer: 8.1 mM Na_2_HPO_4_, 1.76 mM KH_2_PO_4_, 0.137 mM NaCl, 2.683 mM KCl, pH 7.4.

ELISA reaction buffer (1 × PBST): 1 × PBS, 0.05% Tween-20, pH 7.4 (note that Tween-20 was used as the surfactant to improve the solubility of dAA).

Washing buffer: 1 × PBS, pH 7.4.

Blocking buffer: 1 × PBS containing 2% BSA, pH 7.4.

Colour buffer: 53.33 mM HAC-NaAC buffer containing 2.67 mM TMB, 3.33 mM H_2_O_2_, pH 4.5.

### 2.3. Fabrication of Disposable Origami Paper-Based Analytical Device (doPAD)

Large sheets of nitrocellulose filter paper (NC 0.45) and nitrocellulose membrane (HF120) were cut to standard letter sheet size (8.5 × 11 inches) to be fed into the inkjet printer. The proposed doPAD is comprised of two layers. On the first layer, Zone 1 was printed onto a nitrocellulose filter paper using a wax printer (Xerox ColourQube 8570N), followed by heating at 120 °C for 2 min. The wax was melting into the pore structure of cellulose to form the hydrophobic barriers. On the second layer, Zone 2 was printed onto a nitrocellulose membrane using the above wax-printing method. This produced a paper device with a circular zone of diameter 4.5 mm (Zone 1), and a second circular zone of diameter 7 mm (Zone 2). These two layers were assembled together using an adhesive tape. We added 6 μL of cAb (dilution ratio: 1/100; final concentration: 0.06 mg/mL) to Zone 1, followed by incubation at room temperature (RT) for 10 min before washing twice with 100 μL of washing buffer. We then added 25 μL of blocking buffer and incubated it at RT for 25 min. After washing twice in 100 μL of washing buffer, the bioactive paper was dried at RT. We then inkjet-printed 6 μL of 10% pullulan (*w*/*v*) solution on to Zone 1. The doPAD was dried and stored at RT in a desiccant container.

### 2.4. Synthesis of Acrylamide Derivative

We mixed 50 mg of AA, 95 mg of p-mercaptobenzoic acid (4-MPA), 40 mg of NaHCO_3_ and 5 mL of double-distilled water (ddH_2_O) and stirred this in darkness at 37 °C for 90 min. The reaction mixture was then acidified to pH 2.0 with 1 M HCl. After vacuum filtration, the precipitate was washed three times with ddH_2_O. Acrylamide derivatives (dAA) were then obtained after drying, and dissolved in 200 μL of methanol.

### 2.5. Acrylamide Derivatives (dAA) Detection

We mixed 1 μL of dAA (final concentration: 10–5000 μg/L) and 5 μL of HRP-dAA (dilution ratio: 1/2000) and added this to Zone 1. Following incubation at RT for 10 min, Zone 1 was folded on to Zone 2 before adding 20 μL of washing buffer, thus resulting in vertical diffusion of reaction mixtures to Zone 2. This was allowed to proceed for 10 min before adding 15 μL of colour buffer to initiate the colorimetric reaction. JPEG images were taken within ~1 min by a Huawei P30 in a homemade mobile phone holder at a set distance from the paper (about 10 cm high) in the dark. ImageJ software was used to analyze the JPEG images using an 8-bit color scale. The images were then inverted, so that Zone 2, that was originally white, became black. Based on this scale, an increase in the color resulted in an increase in color intensity of the Zone 2.

### 2.6. Specificity

Methylacrylamide, gluotamate, maltose and kanamycin were used as non-target controls. These molecules were first derivatized by 4-MPA according to the procedure for the synthesis of acrylamide derivative, and then subjected to analysis using the identical procedure as described above.

### 2.7. Real Sample Test

Food samples (biscuits, fries) purchased from local supermarkets were dried and homogenized by grinding. 1 g of the obtained samples and 10 mL of 0.1% formic acid/ddH_2_O were added into a 50 mL centrifuge tubes. The tube was then vigorously vortexed for 10 min and centrifuged at 8200× *g* for 10 min at 4 °C [21]. The supernatant was defatted twice with 10 mL of n-hexane [24], followed by passing through 0.22 μM filter membrane. The above solutions were spiked with different concentrations of AA. After being derivatized by 4-MPA, the samples containing dAA were analyzed using doPAD.

## 3. Results and Discussion

### 3.1. Working Principle of doPAD

**Figure 1:**
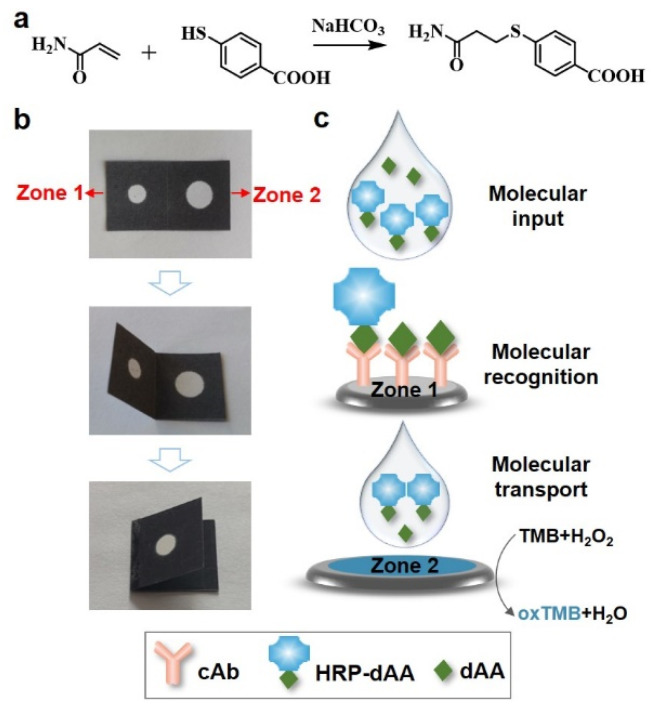
(**a**) Synthesis of acrylamide (AA) derivative, named dAA. (**b**) A foldable disposable origami paper-based analytical device (doPAD), and (**c**) its working principle.

AA derivative, named dAA (Figure 1a and Figure S1), was first synthesized and used as the target for recognizing the coated capture antibody (cAb) on paper. The paper device, illustrated in Figure 1b, consists of two pieces of paper chips folding onto each other. The first piece is configured to be a molecular recognition zone (Zone 1), and the second piece contains a colorimetric readout zone (Zone 2). Zone 1 and Zone 2 are demarcated onto nitrocellulose papers using wax barriers.

To produce a sensor (Figure 1c), Zone 1 was first inkjet-printed with the cAb, blocked by BSA, and then inkjet-printed with a 10% pullulan (*w*/*v*) solution. A transparent pullulan film was obtained after drying at RT. In a typical test, HRP-dAA and dAA in 1 × PBST buffer were simultaneously added to Zone 1. The pullulan films will be dissolved, thus allowing the competitive binding between cAb/HRP-dAA and cAb/dAA. By folding Zone 1 onto Zone 2, any unbound HRP-dAA and dAA are transferred to Zone 2 driven by capillary action. These unbounded HRP-dAA molecules will further oxidize 3,3′,5,5′-tetramethylbenzidine (TMB) in the presence of H_2_O_2_, thus generating a colorimetric readout at Zone 2. In the absence of dAA, no colorimetric signal was produced at Zone 2 because HRP-dAA was expected to bind the coated cAb at Zone 1. Therefore, the color intensity (Zone 2) generated was proportional to the concentrations of dAA. The device could quantify dAA within 21 min, including a target recognition time of 10 min, a vertical diffusion time of 10 min and a colorimetric reaction time of 1 min.

### 3.2. Feasibility of doPAD

**Figure 2:**
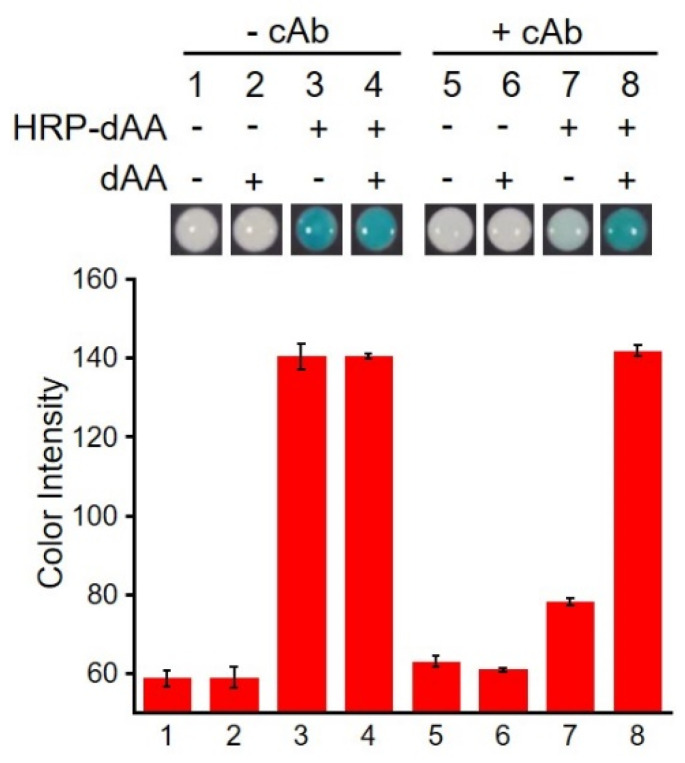
Analysis of dAA by doPAD.

We first investigated the feasibility of performing the colorimetric detection of dAA on paper. In the absence of cAb, a colorimetric signal was produced at the Zone 2 when HRP-dAA was provided both without (Figure 2, lane 3) and with dAA (lane 4), suggesting that HRP-dAA molecules can be easily transported to the Zone 2. In the presence of cAb, we only observed a strong colorimetric signal with the addition of dAA (lane 8). This data indicated that the competitive binding between cAb/HRP-dAA and cAb/dAA at Zone 1, thus resulting in the vertical transport of unbound HRP-dAA to Zone 2. In addition, no significant signal was produced when dAA was omitted (lane 7), suggesting that most HRP-dAA molecules bind the coated cAb at Zone 1.

### 3.3. Optimization of doPAD

**Figure 3:**
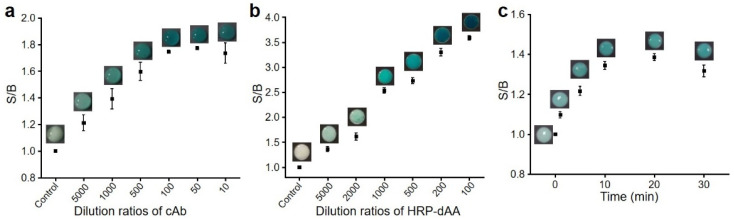
Effects of (**a**) dilution ratios of cAb, (**b**) dilution ratios of horseradish peroxidase-labeled acrylamide derivative (HRP-dAA) and (**c**) incubation time on signal-to-background ratios (S/B).

Many parameters may affect the performance of doPAD. We first optimized the maximum binding capacity of cAb onto Zone 1 (Figure 3a). cAb with certain dilution ratios was first added onto Zone 1 and dried at RT. After blocking with BSA, Zone 1 was washed twice to remove the unbound molecules. With the addition of HRP-dAA, the colorimetric signal produced at Zone 1, defined as the signal-to-background ratio (S/B), gradually increased with decreasing the dilution ratios of cAb from 1/5000 to 1/10. A dilution ratio of 1/100 was used in the following study. We next examined the amounts of HRP-dAA to achieve a low background signal at Zone 2. The key requirement was that HRP-dAA should bind the coated cAb at Zone 1 without the presence of dAA. As shown in Figure 3b, the background signal was decreased at Zone 2 by increasing the dilution ratios of HRP-dAA from 1/100 to 1/2000 at Zone 1. Thus, a dilution ratio of 1/2000 was used to ensure a low background signal. Finally, we performed a kinetic experiment to assess the time required to reach the binding equilibrium between immobilized cAb and HRP-dAA (or dAA). HRP-dAA and dAA were incubated at Zone 1 for different time periods prior to paper folding and analysis. The maximum colorimetric signal was observed when the incubation time was 10 min.

### 3.4. Performance of doPAD

**Figure 4:**
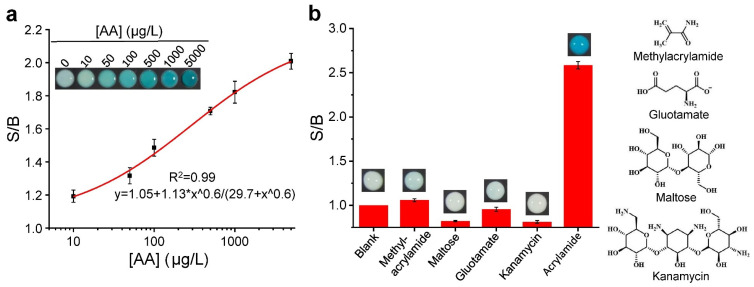
(**a**) Sensitivity and (**b**) specificity of the proposed doPAD.

We utilized this proposed doPAD for the detection of AA. Figure 4a shows the images of colorimetric responses of Zone 2 as a function of AA concentration. The response curve was obtained by measuring the color intensity using ImageJ for each paper well array. It was observed that the S/B values gradually increased with increasing AA concentrations. A detection limit of 1.13 μg/L was obtained on the basis of the 3 s/slope (s, standard deviation of the blank samples).

Besides the high sensitivity, each doPAD also exhibited excellent selectivity for its cognate target (Figure 4b). No obvious colorimetric signal was observed when each system was tested with other unintended small compounds: methylacrylamide, gluotamate, maltose and kanamycin.

We next evaluate the stability and the reproducibility of the doPADs. A group of devices were stored at RT in the dark and tested over a period of 15 days. The devices remained at approximately 80% of their initial activity (Figure S2). When comparing between 30 individual paper sensors, a coefficient of variation of 8.2% was obtained, indicating the good reproducibility of this device (Figure S3).

**Figure 5:**
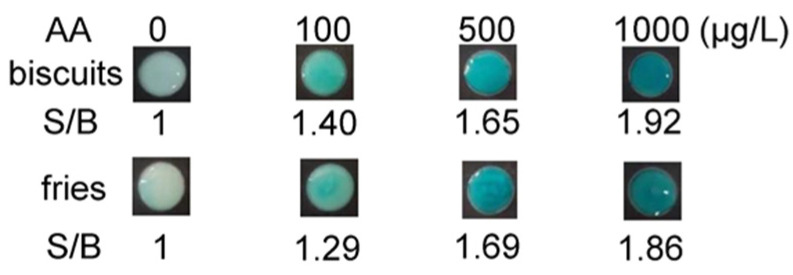
Detection of AA in the presence of complex sample matrices represented by biscuits and fries.

We also challenged the doPAD by analyzing AA spiked in complex food matrices represented by biscuits and fries (Figure 5). This experiment shows that background materials in these complex samples do not significantly affect the outcome of the results. A strong colorimetric signal was observed upon addition of the target, producing S/B values that were proportional to the spiked AA concentration. One observed that the developed doPAD displayed recoveries ranging from 82% to 106% for the food samples along with a relative standard deviation (RSD) below 8% (Table 1). This result suggested the potential applicability of our assay for the quantification of AA in real food samples.

## 4. Conclusions

In summary, we have demonstrated a cost-effective, integrated paper-based analytical device for colorimetric detection of AA in food products. The device features three components: a capture antibody for its ability to selectively recognize the small molecule on paper, origami paper for the precise control of the fluidic flow, and HRP for its ability to amplify each recognition event into colorimetric signals that can be easily detected. The detection limit of 1.13 μg/L was achieved within 120 min. The advantage of the sensitive and rapid analysis may help for in-time detection of toxic chemicals in the field of food safety. Although we have provided illustrative examples for detecting acrylamide in food samples, this approach could be easily extended to other types of small molecules. Future work will include integration of the sample extraction module with this paper-based device to increase its portability and usability in remote settings.

## Figures and Tables

**Table 1:** Recovery (%) and relative standard deviation (RSD, %) of the proposed doPAD in the detection of AA with different concentrations.

Samples	Added (μg/L)	Recovery (%)	RSD (%)
biscuits	100	82–95	7.14
	500	95–106	5.42
	1000	96–105	5.22
fires	100	85–94	5.31
	500	96–106	7.27
	1000	95–104	6.16

## Supplementary Materials

The following supporting information can be downloaded at https://www.mdpi.com/article/10.3390/mi13010013/s1, Figure S1: HR-MS of dAA; Figure S2: stability; Figure S3: reproducibility.
