# Supplementary material for: An Origami Paper-Based Analytical Device for Rapid and Sensitive Analysis of Acrylamide in Foods
Source: Micromachines (Basel). 2021 Dec 23;13(1):13. doi: 10.3390/mi13010013 (PMC8777769; doi:10.3390/mi13010013)
Supplement: Supplementary file 1 [file micromachines-13-00013-s001.zip › micromachines-1488983-supplementary.pdf]

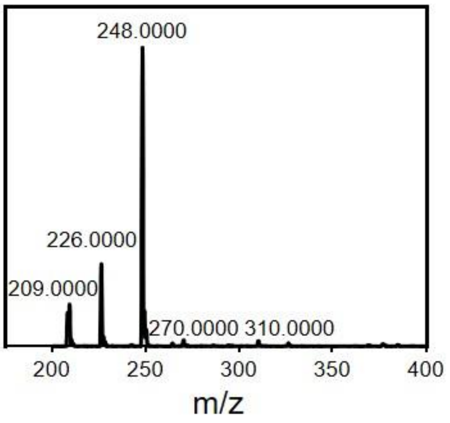

Figure S1. HR-MS of dAA. calcd. for $C_{10}H_{11}NNaO_3S^+$ $[M+Na]^+$, 248.0352; found, 248.0000; calcd. for $C_{10}H_{12}NO_3S^+$ $[M+H]^+$, 226.0089; found, 226.0000.

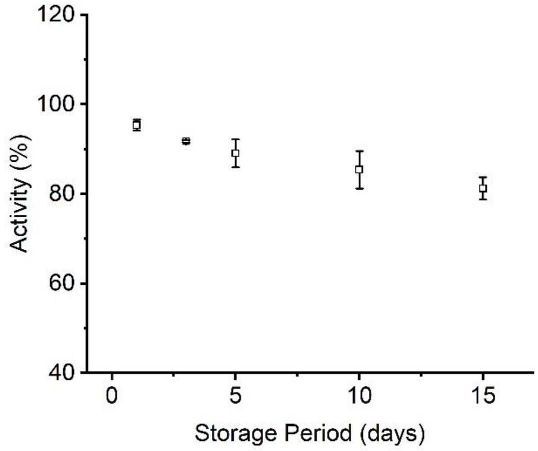

Figure S2. Evaluation of the stability of the doPADs stored at room temperature.

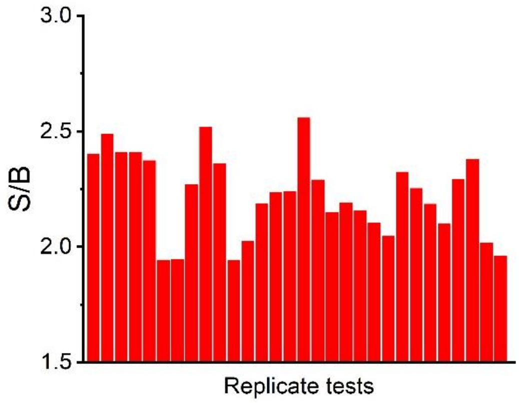

Figure S3. Evaluation of the reproducibility of the doPADs.
